# Effects of salt stress on root morphology, carbon and nitrogen metabolism, and yield of Tartary buckwheat

**DOI:** 10.1038/s41598-023-39634-0

**Published:** 2023-08-01

**Authors:** Xinyue Zhang, Peiyun He, Rongyu Guo, Kaifeng Huang, Xiaoyan Huang

**Affiliations:** School of Life Science, Guizhou Normal University, No.116 Baoshan North Road, Guiyang, 550001 People’s Republic of China

**Keywords:** Plant sciences, Environmental sciences

## Abstract

This study aims to clarify the effects of different concentrations of sodium chloride on the carbon and nitrogen metabolism and yield of Tartary buckwheat. The salt-sensitive cultivar Yunqiao 2 was pot-grown and treated with four salt concentrations including 0, 2, 4, and 6 g kg^−1^. The root morphology index increased from seedling stage to maturate stage. The content of soluble protein in the leaves reached the maximum at the anthesis stage, and the other substances content and the enzymes activity related to carbon and nitrogen metabolism reached the maximum at the grain filling stage. The root morphology index, root activity; invertase, amylase, sucrose synthase, and sucrose phosphate synthase activities; nitrate-nitrogen, ammonium nitrogen, and soluble protein content; and nitrate reductase and glutamate synthase activities increased first and reached the maximum at 2 g kg^−1^ treatment and then decreased with increasing salt stress concentration. The content of soluble sugars and sucrose and the activity of glutamate dehydrogenase increased continuously with increasing salt concentration, and reached the maximum in the 6 g kg^−1^ treatment. The grain number per plant, 100-grain weight, and yield per plant increased first and reached the maximum at 2 g kg^−1^ treatment and then decreased with increasing salt stress concentration. In summary, moderate salt stress (2 g kg^−1^) can promote the root growth, increase the content of carbon and nitrogen metabolism-related substances and enzyme activity, and increase the yield per plant of Tartary buckwheat.

## Introduction

Saline-alkali soil in China has an extensive distribution. The total area of saline soil is 3.69 × 10^7^ hm^2^, accounting for about 5% of the total cultivated land area in China. The area of saline soil ranks third in the world, and the area of saline soil are still increasing year by year^1^. Soil salinization is one of the major types of abiotic stress limiting agricultural development^2^. The excessive accumulation of sodium chloride (NaCl) in saline soil result in osmotic stress, ion toxicity, and oxidative stress in plants, resulting in a sharp decrease in seed germination, chlorophyll degradation, and photosynthetic rates and changes in osmotic pressure. The absorption of salt by transporters from soil induces ion toxicity and disrupts ion absorption and its metabolic balance, thus affecting the normal growth of plants^3–5^.

Nitrogen and carbon metabolism are the two most important metabolic processes in plants and play a vital role in the whole life activities of plants^6,7^. Nitrogen is one of the basic nutrients required for plant growth and development^8^. It is the main component of amino acids and proteins and can regulate nitrogen assimilation and carbohydrate metabolism. Plants use nitrogen in the form of nitrate and ammonium, which are converted into various amino acids^9^. Apart from the legume family, higher plants fix nitrogen by first reducing its inorganic form NO_3_^−^ to NH_4_^+^ and then incorporating it into an organic form^10,11^. This process mainly occurs through the glutamine synthetase/glutamate synthase (GS/GOGAT) cycle^12,13^. Nitrogen metabolism is regulated by external factors, such as heat, drought, and salinity^14^. Salt stress inhibits nitrogen uptake and metabolism, especially nitrate uptake by plants^15^. Sha^15^ found that nitrogen content in rice leaves and sheath decreases significantly under salt stress, nitrate-nitrogen content decreases, and ammonium nitrogen content increases. Yang^16^ found that nitrate reductase (NR) activity decreases with nitrate content in plants under salt stress. Lutts et al.^17^ found that salt stress significantly reduces the activity of GS in rice leaves and the GS activity of salt-sensitive varieties is lower than that of salt-tolerant varieties.

In plants, carbon metabolism is the most important and basic physiological metabolism, which is of great significance to plant growth and development, transformation of various chemical components, and final yield^8^. Invertase, sucrose synthase (SS), sucrose phosphate synthase (SPS), and amylase are important enzymes in the carbon metabolism pathway of plants, and their activities directly affect the accumulation of carbohydrates in plants. Invertase is a hydrolase that can convert sucrose into monosaccharide and facilitate the remobilization of sugar in plants. A series of glucose metabolism disorders occurs in plants under stress. Salt stress has significant effects on carbon metabolism in plants. Peng et al.^18^ reported that salt stress increases the activities of SS and SPS in cotton leaves. The content of sucrose and fructose in plant cells increases with salt concentration^19^. Strong salt-tolerant wheat alleviates salt stress by synthesizing large amounts of soluble sugars and soluble protein^20^. Salt stress promotes the synthesis and accumulation of soluble sugars in leaves of different salt-tolerant rice cultivars^21^.

Tartary buckwheat (*Fagopyrum tataricum* Gaertn) belongs to genus *Fagopyrum* Mill. It has remarkable functions, such as lowering blood sugar, blood pressure, and blood lipid level^22^ and has great market demand globally, especially in Europe, America, and Southeast Asia^23^. To date, research on Tartary buckwheat and salt stress mainly focuses on seed germination^24^, seedling physiological characteristics^25^, genomics and transcriptomics^26^. The effects of salt stress on the carbon and nitrogen metabolism of Tartary buckwheat are rarely explored. Therefore, this experiment used the salt-sensitive Tartary buckwheat variety Yunqiao 2 as the experimental material in studying the effects of salt stress on the root morphology and physiology, leaf carbon and nitrogen metabolism, and yield formation of Tartary buckwheat to reveal the relationship between the salt tolerance and carbon and nitrogen metabolism of Tartary buckwheat and provide a reference for the high-yield cultivation of Tartary buckwheat.

## Materials and methods

### Plant materials and growing condition

The seeds of salt-sensitive Tartary buckwheat variety Yunqiao 2 used in this study were collected from the Yunnan Academy of Agricultural Sciences, China (25° 05′ N, 102° 71′ E), and complied with relevant institutional, national, and international guidelines and legislation. We have obtained the permission to collect seeds. The experiment was conducted from September to November 2021 at the growth chamber and outdoor potting test site of the Research Center of Buckwheat Industry Technology of Guizhou Normal University (106° 27′ E, 26° 44′ N). A canopy was built on top of the outdoor potted plant test site to prevent rain. The monthly average temperatures and monthly average sunshine from September to November were 16.9 °C and 193.8 h in 2021 (data obtained from the Guiyang Meteorological Bureau website of China; http://gz.cma.gov.cn/dsqxj/gy/index.html; accessed on May 5, 2022). The walls and roof of the growth chamber were made of translucent polyurethane material. Sunlight was projected, and temperature could not be controlled. The monthly average temperature from September to November of the growth chamber was 17.0 °C and the average relative humidity was 50.0%.

### Treatment

New soil (pH 6.29) containing 10.82 g kg^−1^ organic matter, 2.97 mg kg^−1^ available nitrogen, 14.71 mg kg^−1^ available phosphorus, and 103.76 mg kg^−1^ available potassium was collected from the experiment station. Soil nutrient contents were determined using a multichannel intelligent soil nutrient meter (OK-V24, China).

According to the quality of soil in the porcelain pot, sodium chloride (NaCl) was added for the preparation of low-salt (S2), medium-salt (S4), and high-salt (S6) soil with salt content of 2, 4, and 6 g kg^−1^, respectively. New soil without NaCl was used as the control (CK, NaCl 0 g kg^−1^). Each porcelain pot (25 cm in diameter and 18 cm in height) was filled with 5 kg of soil with a different salt concentration.

On September 8, 2021, the seeds of Tartary buckwheat with the same size were selected and disinfected with 1.2% HgCl_2_ for 30 min, washed with distilled water, and air-dried, and sowed. Fifteen seeds were sowed in each porcelain pot, and 10 seedlings were set after emergence. Instead of compound fertilizer (N: P: K = 15:15:15) dosage of 600 kg ha^−1^ (usually used by local farmers), 2.9 g of compound fertilizer as the base fertilizer was applied once per pot before sowing and fully mixed with saline soil. Fertilization was not performed in the whole growth period. Every 10 days, 1.5 L of water was added to each porcelain pot, and leaching caused by salt reduction was prevented by returning water from the bottom tray to the porcelain pot immediately. Each treatment was performed on 20 porcelain pots.

### Sampling and measurements

#### Root morphology and root activity

Five Tartary buckwheat plants with similar growth were randomly selected from porcelain pots treated in the growth chamber and outdoor pot experiment field at the seedling stage (19 days after sowing), flowering stage (40 days after sowing), grain filling stage (55 days after sowing), and maturation stage (outdoor, 77 days after sowing; growth chamber, 80 days after sowing), and the whole plants were dug. After carefully scooping up soil near the plant with a shovel, the plant was gently pulled up, and the integrity of the root system was preserved as much as possible. After washing with clean water, the Tartary buckwheat roots were cut. After surface water in the roots was removed with absorbent paper, all roots were image-scanned using a root scanner (GXY-A, Zhejiang Tuopu Instrument Co., Ltd.)^27^. Root length, surface area, volume, and mean diameter were obtained with the root analysis system WinRHIZO (version 4.0b, Regent Instruments Inc.). Root activity was determined using the 2,3,5-triphenyl-tetrazolium chloride method^28^.

#### Substance content and enzyme activity related to carbon and nitrogen metabolism

At the seedling stage, flowering, grain filling, and mature stages, fresh leaves on the one to four sections at the top of the main stem of Tartary buckwheat were collected from the growth chamber and outdoor potting test field, respectively. Some of the leaves were frozen in liquid nitrogen for 30 s and quickly stored in a refrigerator at − 80 °C. The other part was deactivated at 105 °C for 15 min, dried to constant weight in an oven at 80 °C, crushed, passed through a 40-mesh sieve, and stored.

The content of soluble sugars was determined by anthrone colorimetry. 0.2 g of dry Tartary buckwheat leaves was weighed, ground evenly with 4 mL of 80% ethanol, and kept in a water bath at 80℃ for 30 min. After centrifugation at 5000 r/min for 10 min, the supernatant was transferred, and the residue was added with 2 mL of 80% ethanol for repeated extraction. Then, the supernatant was combined. The activated carbon was added, decolored in a water bath for 30 min, diluted to 10 mL, and filtered to obtain soluble sugars and sucrose solution. 0.5 mL of the solution to be measured was mixed with an equal volume of distilled water, and 5 mL of anthrone sulfate solution was added to shake quickly. The mixture was boiled for 10 min, and the light absorption value at 620 nm was measured after cooling^28^.

The sucrose content was determined by using the resorcinol method. 0.4 mL of the above mentioned test solution and 0.2 mL of 2 mol/L NaOH were collected and boiled in water for 5 min. After cooling, 2.8 mL of 30% HCl and 0.8 mL of 0.1% resorcinol reagent were added. The mixture was shaken, mixed, and kept at 80℃ for 10 min. The optical density value at 480 nm was measured after cooling^28^.

The method of He^29^ was used to determine invertase activity. 0.5 g of fresh Tartary buckwheat leaves was ground with pre-cooled distilled water and diluted to 10 mL. After centrifugation at 4000 r/min for 15 min, the supernatant was collected for analysis. After mixing 1 mL of crude enzyme solution, 2.5 mL of pH6.0 phosphate-buffered saline, and 0.5 mL of 10% sucrose solution, the mixture was incubated in a water bath at 37 °C for 0.5 h. One milliliter of the above mentioned mixture was collected and added with 0.75 mL of 3,5-dinitrosalicylic acid, and the mixture was boiled for 5 min. After cooling in an ice-water bath, the mixture was diluted to 10 mL with distilled water, and the OD value at 540 nm was determined.

The SS and SPS activities were determined in accordance with the method of Guo^30^. 0.5 g of fresh Tartary buckwheat leaves was ground evenly with 3 mL of Hepes–NaOH extraction buffer and centrifuged at 10000 r/min for 10 min. The supernatant was taken as the enzyme solution to be tested. The reaction mixture system 50 μL of Hepes–NaOH buffer (pH7.5), 20 μL of 0.05 mol/L MgCl_2_, 20 μL of 0.1 mol/L UDPG, 20 μL of 0.1 mol/L fructose, and 50 μL of crude enzyme extract. The control was replaced with the inactivated enzyme solution. The above mentioned mixture was bathed in water at 30 °C for 0.5 h, and 0.2 mL of 2 mol/L NaOH solution was added to terminate the reaction. The mixture was bathed in boiling water for 10 min. After cooling, 1.5 mL of 30% HCl and 0.5 mL of 0.1% resorcinol were added, and the mixture was bathed in water at 80 °C for 10 min. The optical density value at 480 nm was recorded.

The extraction method of SPS activity was the same as that of SS. In the reaction mixture system, fructose 6-phosphate was used instead of fructose, and the other methods were the same as those of SS.

Amylase activity was determined in accordance with the method of Tang and Luo^28^. First, 2–5 g of fresh leaves was collected, added with 2–4 mL of pH5.6 citric acid buffer in the mortar, ground into homogenate, added with 6 mL of buffer, transferred to a 50 mL volumetric flask, and extracted in a 40 °C water bath for 1 h. Afterward, the mixture was shaken, stand, and centrifuged. Ten millimeters of the supernatant was absorbed. Subsequently, 2 mL of 8% oxalic acid solution was added to each tube and bathed in boiling water for 15 min. Then, two drops of 0.1% phenolphthalein reagent were added to each tube and neutralized with 1 mol · L^−1^ NaOH solution at a constant volume of 25 mL. One milliliter of the aforementioned reagent was added into a 10 mL test tube, added with 1 mL of DNS reagent, boiled for 5 min, and quickly cooled.Then, distilled water was used to scale, and the OD value was measured at a wavelength of 520 nm.

The content of nitrate nitrogen was determined using the method of Tang and Luo^28^. Three milliliters of distilled water was added to a mortar containing 0.5 g of fresh Tartary buckwheat leaves for grinding. The grinding solution was extracted in a 100 °C water bath for 0.5 h, and the volume was adjusted to 5 mL after filtration. 0.4 mL of salicylic acid–sulfuric acid solution was added to 0.1 mL of test solution. The mixture was stand for 20 min and added with 9.5 mL of 0.08 g/mL NaOH solution. The light absorption value was measured at a wavelength of 410 nm.

The content of ammonium nitrogen was determined using the method of Tang and Luo^28^. 0.5 g of fresh Tartary buckwheat leaves was ground into a homogenate with 5 mL of 10% acetic acid solution, diluted with distilled water to 100 mL, and filtered for later use. Two milliliters of the test solution was added with 3 mL of ninhydrin hydrate and 0.1 mL of 1% ascorbic acid and heated at 100 °C for 15 min. After cooling, the liquid was diluted to 10 mL with anhydrous ethanol, and the optical density value was measured at OD580 nm after shaking.

The soluble protein content was determined by using the Coomassie brilliant blue method^28^. 0.3 g of fresh Tartary buckwheat leaves was added to 5 mL of distilled water, ground, and centrifuged at 3000 r/min for 10 min. 0.5 mL of the supernatant and equal volume of distilled water were added to 5 mL of Coomassie brilliant blue solution, and the light absorption value was measured at 595 nm after 2 min.

The activity of NR was determined in accordance with the method of Tang and Luo^28^. 0.5 g of fresh Tartary buckwheat leaves was ground with 4 mL of extract and centrifuged at 4000 r/min for 15 min. The supernatant contained the test solution. 0.4 mL of enzyme solution was added to 1.2 mL of 0.1 mol/L KNO_3_ phosphate buffer and 0.4 mL of NADH solution, and the solution was bathed in water at 25 °C for 0.5 h. One milliliter of sulfonamide solution was added to terminate the reaction, and then 1 mL of naphthyl vinylamine was added to develop the color for 15 min. After centrifugation, the supernatant was collected, and the OD value was measured at 540 nm.

GOGAT activity was determined in accordance with the method of Yu^31^. The extraction method of crude enzyme solution was the same as that of GS. The reaction mixture consisted of 1.5 mL of Tris–HCl buffer (pH 7.6), 0.1 mL of 10 mmol/L KCl, 0.5 mL of 20 mmol/L α-ketoglutarate, 0.2 mL of 3 mmol/L NADH, 0.3 mL of enzyme solution, and 0.4 mL of 20 mmol/L L-glutamine. After the reaction was started, the change in OD value was recorded every 20 s at 340 nm for 10 consecutive times, and the change in enzyme activity was measured by decreasing the optical density value.

The activity of glutamate dehydrogenase was determined in accordance with the method of Loulakakis and Roubelakis-Angelakis^32^. The extraction method of crude enzyme solution was the same as that of GS. The reaction system contained 2.6 mL of crude enzyme solution, 0.1 mL of ddH_2_O, 0.1 mL of 30 mmol/L CaCl_2_, 0.1 mL of 6 mmol/L NADH, and 0.1 mL of crude enzyme extract. The change in OD value was measured at 340 nm. The OD value was recorded every 20 s and continuously measured 10 times. A section with a stable decrease in OD value was taken to measure the change in enzyme activity.

#### Yield

Grains were harvested from each porcelain plot when at least 70–80% of the grains were mature (Outdoor, November 24; growth chamber, November 27). After the seeds were naturally dried, the number of grains per plant and yield per plant were determined, and the average of three replicates was calculated^7,27^.

#### Statistical analyses of experimental data

Data were processed using Microsoft Excel 2003 and SPSS 22.0. One-way ANOVA was performed, and means were tested by least significant difference at *P* = 0.05 (LSD 0.05). The results of the growth chamber and outdoor potting test site were similar. Therefore, the outdoor potting test site data were presented in this study, and the data from the growth chamber were deposited as supplementary data.

## Results

### Effects of salt stress on root morphology and root activity

The root length, root surface area, and root volume of Tartary buckwheat were increased from seedling stage to maturity stage (except the root length of S4 and S6 treatments; Table 1). The average root diameter reached a peak at the grain filling stage. Root activity decreased gradually with growth period. The root length, root surface area, root volume, average root diameter, and root activity in the S6 treatment were the lowest. Compared with CK, the S2 treatment increased the root length, root surface area, root volume, average root diameter, and root activity by 6.41%, 13.04%, 8.33%, 15.36%, and 17.50%, respectively.

**Table 1:** Effects of salt stress on root morphology and root activity of Tartary buckwheat.

Item	Treatment	Seedling stage	Flowering stage	Grain filing stage	Maturate stage
Root length (cm)	CK	22.96 ± 1.70b	46.43 ± 2.22a	82.25 ± 2.73a	95.37 ± 4.12a
	S2	31.48 ± 1.23a	47.22 ± 3.72a	86.97 ± 3.98a	97.17 ± 2.26a
	S4	16.82 ± 2.57c	44.21 ± 4.06a	36.72 ± 2.36b	29.38 ± 2.80b
	S6	15.03 ± 1.44c	41.49 ± 3.04b	32.19 ± 2.07b	25.90 ± 1.88b
Root surface area (cm^2^)	CK	4.61 ± 0.36a	7.05 ± 0.18b	15.23 ± 1.35a	20.50 ± 1.91a
	S2	5.45 ± 0.16a	10.13 ± 0.85a	16.70 ± 0.79a	21.29 ± 1.15a
	S4	3.68 ± 0.17b	6.94 ± 0.59b	14.49 ± 1.81b	19.15 ± 1.26b
	S6	3.03 ± 0.35b	6.16 ± 0.15b	13.82 ± 1.56b	18.30 ± 1.77b
Root volume (cm^3^)	CK	0.29 ± 0.03a	0.92 ± 0.13a	1.71 ± 0.09a	3.56 ± 0.14a
	S2	0.34 ± 0.02a	1.05 ± 0.07a	1.80 ± 0.13a	3.83 ± 0.12a
	S4	0.27 ± 0.02a	0.58 ± 0.04b	1.53 ± 0.13b	2.93 ± 0.11b
	S6	0.26 ± 0.02a	0.49 ± 0.07b	1.47 ± 0.16b	2.77 ± 0.12b
Average diameter (mm)	CK	0.65 ± 0.04a	0.73 ± 0.04b	0.93 ± 0.07b	0.88 ± 0.09a
	S2	0.76 ± 0.05a	0.95 ± 0.04a	1.02 ± 0.06a	0.95 ± 0.04a
	S4	0.56 ± 0.06a	0.64 ± 0.05b	0.71 ± 0.05b	0.65 ± 0.06b
	S6	0.49 ± 0.04b	0.57 ± 0.06b	0.68 ± 0.06b	0.59 ± 0.07b
Root activity (μg (gh)^−1^)	CK	6.75 ± 0.04b	6.47 ± 0.33a	2.34 ± 0.23a	2.10 ± 0.15a
	S2	8.65 ± 0.60a	6.96 ± 0.25a	2.76 ± 0.25a	2.38 ± 0.26a
	S4	6.50 ± 0.35b	6.33 ± 0.34a	1.97 ± 0.29b	0.97 ± 0.11b
	S6	6.04 ± 0.17b	5.85 ± 0.56a	1.86 ± 0.21b	0.82 ± 0.08b

Different letters indicate statistical significance at the *P* < 0.05 level within the same column.

CK, Control (0 g kg^−1^); S2, Low-salt (2 g kg^−1^); S4, Medium-salt (4 g kg^−1^); S6, High-salt (6 g kg^−1^).

### Effects of salt stress on contents of carbon metabolism related substances

The content of soluble sugars and sucrose in the leaves of Tartary buckwheat reached the maximum at the grain filling stage and the minimum at the seedling stage (Table 2). The content of soluble sugars and sucrose in leaves increased continuously with increasing salt concentration. Compared with CK, the S6 treatment increased the soluble sugars and sucrose content by 1.36 and 1.40 times.

**Table 2:** Effects of salt stress on carbon metabolism related substances in Tartary buckwheat.

Item	Treatment	Seedling stage	Flowering stage	Grain filing stage	Maturate stage
Soluble sugars (mg g^−1^)	CK	66.24 ± 3.70d	104.53 ± 4.97d	231.53 ± 6.52d	117.92 ± 9.31d
	S2	74.33 ± 3.31c	125.36 ± 5.85c	247.26 ± 11.01c	134.84 ± 8.21c
	S4	85.63 ± 5.68b	140.36 ± 8.31b	259.37 ± 13.21b	155.28 ± 9.87b
	S6	94.90 ± 3.88a	163.50 ± 10.9a	273.89 ± 14.7a	173.02 ± 7.49a
Sucrose (mg g^−1^)	CK	30.55 ± 2.01d	48.32 ± 1.75d	155.48 ± 6.77d	44.40 ± 1.57d
	S2	39.71 ± 2.14c	64.65 ± 3.42c	167.61 ± 10.59c	50.38 ± 3.51c
	S4	45.92 ± 2.34b	76.34 ± 3.83b	175.81 ± 5.97b	55.67 ± 4.46b
	S6	51.16 ± 2.65a	89.38 ± 3.06a	182.84 ± 8.61a	66.48 ± 3.94a

Different letters indicate statistical significance at the *P* < 0.05 level within the same column.

CK, Control (0 g kg^−1^); S2, Low-salt (2 g kg^−1^), S4, Medium-salt (4 g kg^−1^); S6, High-salt (6 g kg^−1^).

### Effects of salt stress on enzyme activities related to carbon metabolism

The activities of invertase, amylase, SS, and SPS in the leaves reached a peak at the grain filling stage (Table 3). The activities of invertase, amylase, SS, and SPS in the S2 treatment were 1.02, 1.08, 1.07, and 1.09 times higher than those of CK. The leaves under each treatment were sequenced according to enzyme activities as follows: S2 > CK > S4 > S6.

**Table 3:** Effects of salt stress on enzyme activities related to carbon metabolism in Tartary buckwheat.

Item	Treatment	Seedling stage	Flowering stage	Grain filing stage	Maturate stage
Invertase (mg g^−1^ h^−1^)	CK	4.83 ± 0.12b	8.89 ± 0.60b	17.58 ± 1.07b	9.46 ± 0.21b
	S2	5.00 ± 0.31a	9.00 ± 0.44a	17.70 ± 1.41a	9.75 ± 0.14a
	S4	4.58 ± 0.37c	8.84 ± 0.57c	17.39 ± 0.82c	9.23 ± 0.22c
	S6	4.27 ± 0.21d	8.61 ± 0.32d	17.10 ± 1.08d	9.01 ± 0.30d
Amylase (mg g^−1^ h^−1^)	CK	1.39 ± 0.09b	2.90 ± 0.09b	6.96 ± 0.18b	2.93 ± 0.12b
	S2	1.51 ± 0.10a	3.11 ± 0.10a	7.43 ± 0.28a	3.21 ± 0.19a
	S4	1.31 ± 0.08b	2.69 ± 0.18c	6.81 ± 0.20b	2.89 ± 0.22b
	S6	1.29 ± 0.11c	2.47 ± 0.13d	6.46 ± 0.37c	2.49 ± 0.18c
Sucrose synthase (SS, mg g^−1^ h^−1^)	CK	17.26 ± 1.04b	26.09 ± 1.07b	40.45 ± 1.51b	32.93 ± 2.69b
	S2	18.99 ± 1.48a	27.90 ± 1.69a	42.54 ± 2.17a	35.02 ± 1.90a
	S4	14.60 ± 1.93c	23.23 ± 1.22c	37.75 ± 2.48c	31.12 ± 1.78c
	S6	13.89 ± 0.90d	20.25 ± 1.01d	35.22 ± 2.71d	30.50 ± 1.99c
Sucrose phosphate synthase (SPS, mg g^−1^ h^−1^)	CK	12.98 ± 1.03b	21.34 ± 1.77b	34.55 ± 2.53b	28.06 ± 1.81b
	S2	15.08 ± 1.06a	23.18 ± 1.81a	37.19 ± 2.62a	30.25 ± 1.65a
	S4	9.71 ± 0.11c	19.87 ± 1.61c	32.52 ± 2.54c	26.13 ± 1.42c
	S6	8.38 ± 0.29d	15.52 ± 1.32d	30.67 ± 1.98d	24.21 ± 1.39d

Different letters indicate statistical significance at the *P* < 0.05 level within the same column.

CK, Control (0 g kg^−1^), S2, Low-salt (2 g kg^−1^), S4, Medium-salt (4 g kg^−1^), S6, High-salt (6 g kg^−1^).

### Effects of salt stress on contents of nitrogen metabolism related substances

The content of nitrate-nitrogen and ammonium nitrogen reached the maximum at the grain filling stage, and the content of soluble protein reached a peak at the anthesis stage (Table 4). The S6 treatment had the lowest content of nitrate-nitrogen, ammonium nitrogen, and soluble protein. Compared with CK, the S2 treatment increased the nitrate-nitrogen, ammonium nitrogen, and soluble protein content by 5.84%, 6.65%, and 2.20%, respectively.

**Table 4:** Effects of salt stress on nitrogen metabolism related substances in Tartary buckwheat.

Item	Treatment	Seedling stage	Flowering stage	Grain filing stage	Maturate stage
Nitrate nitrogen (mg g^−1^)	CK	84.64 ± 2.49b	117.87 ± 4.33b	148.37 ± 7.75b	102.83 ± 6.27a
	S2	92.57 ± 2.36a	125.80 ± 6.19a	155.32 ± 9.09a	106.52 ± 6.01a
	S4	82.76 ± 2.75b	112.67 ± 3.13c	141.29 ± 10.75c	94.53 ± 5.28b
	S6	76.69 ± 2.34c	106.33 ± 5.69d	135.28 ± 11.04d	86.70 ± 5.53c
Ammonium nitrogen (mg g^−1^)	CK	101.47 ± 6.26b	114.80 ± 3.48b	133.12 ± 9.55b	98.57 ± 6.20a
	S2	109.23 ± 5.69a	124.72 ± 5.16a	141.72 ± 8.20a	102.08 ± 5.57a
	S4	87.54 ± 2.17c	106.86 ± 4.52c	119.16 ± 6.69c	91.33 ± 4.76b
	S6	74.44 ± 3.39d	89.71 ± 3.34d	105.33 ± 5.66d	86.78 ± 4.24c
Soluble protein (mg g^−1^)	CK	18.51 ± 0.94b	72.93 ± 1.81b	59.91 ± 2.27b	46.87 ± 1.30b
	S2	19.30 ± 0.92a	73.84 ± 1.56a	61.47 ± 2.15a	47.97 ± 1.28a
	S4	18.02 ± 0.74bc	72.02 ± 1.46c	59.41 ± 2.22bc	46.20 ± 1.09bc
	S6	17.80 ± 0.88c	71.14 ± 1.58d	58.03 ± 1.30c	45.36 ± 1.05c

Different letters indicate statistical significance at the *P* < 0.05 level within the same column.

CK, Control (0 g kg^−1^), S2, Low-salt (2 g kg^−1^), S4, Medium-salt (4 g kg^−1^), S6, High-salt (6 g kg^−1^).

### Effects of salt stress on activities of nitrogen metabolism related enzymes

The activities of NR, GOGAT, and GDH in the leaves reached a peak at the grain filling stage (Table 5). Compared with CK, the S2 treatment increased the NR and GOGAT activities by 1.61% and 2.10%, respectively. The S6 treatment had the highest GDH activity.

**Table 5:** Effects of salt stress on enzyme activities related to nitrogen metabolism in Tartary buckwheat.

Item	Treatment	Seedling stage	Flowering stage	Grain filing stage	Maturate stage
Nitrate reductase (NR, mg g^−1^ h^−1^)	CK	14.11 ± 0.90b	45.95 ± 2.67ab	71.24 ± 1.65b	23.15 ± 1.14b
	S2	14.80 ± 0.88a	46.25 ± 1.04a	71.96 ± 1.93a	23.92 ± 1.47a
	S4	13.89 ± 0.91b	45.33 ± 1.74b	70.88 ± 1.86c	22.77 ± 1.79bc
	S6	13.27 ± 0.86c	44.93 ± 2.75c	70.15 ± 1.42d	22.18 ± 1.14c
Glutamate synthase (GOGAT, mg g^−1^ h^−1^)	CK	13.94 ± 0.96b	45.89 ± 2.37b	68.75 ± 1.86b	24.02 ± 1.88b
	S2	14.20 ± 0.59a	46.76 ± 2.56a	69.85 ± 1.73a	24.99 ± 1.98a
	S4	13.40 ± 0.88c	45.51 ± 1.82bc	68.04 ± 1.02c	23.85 ± 1.71b
	S6	12.90 ± 0.93d	45.23 ± 1.02c	67.89 ± 1.78c	23.15 ± 1.58c
Glutamate dehydrogenase (GDH, mg g^−1^ h^−1^)	CK	14.32 ± 0.64b	24.78 ± 1.39c	27.01 ± 0.80d	14.27 ± 0.65d
	S2	14.57 ± 0.85b	25.65 ± 1.14b	27.82 ± 1.05c	14.84 ± 0.86c
	S4	15.03 ± 0.48ab	25.87 ± 1.36b	28.72 ± 0.98b	15.76 ± 0.81b
	S6	15.43 ± 0.91a	26.66 ± 1.05a	29.74 ± 1.62a	16.40 ± 0.69a

Different letters indicate statistical significance at the *P* < 0.05 level within the same column.

CK, Control (0 g kg^−1^), S2, Low-salt (2 g kg^−1^), S4, Medium-salt (4 g kg^−1^), S6, High-salt (6 g kg^−1^).

### Effect of salt stress on the yield of Tartary buckwheat

The yield per plant of the S2 treatment was significantly higher than the yield of the CK, S4, and S6 treatments (Table 6). The 100-grain weight and grain number per plant of the S2 and CK treatments were significantly higher than those of the S4 and S6 treatments. Compared with CK, the S2 treatment increased the number of grains per plant, 100-grain weight, and yield per plant by 7.06%, 2.13%, and 3.16%, respectively.

**Table 6:** Effects of salt stress on yield of Tartary buckwheat.

Treatment	Grain number per plant	Hundred grain weight (g)	Grain weight per plant (g)
CK	28.33 ± 0.99a	2.38 ± 0.17ab	0.36 ± 0.01b
S2	30.33 ± 1.36a	2.43 ± 0.14a	0.38 ± 0.02a
S4	24.33 ± 0.96b	2.30 ± 0.10b	0.35 ± 0.01c
S6	22.33 ± 1.05b	2.30 ± 0.11b	0.34 ± 0.02d

Different letters indicate statistical significance at the *P* < 0.05 level within the same column.

CK, Control (0 g kg^−1^), S2, Low-salt (2 g kg^−1^), S4, Medium-salt (4 g kg^−1^), S6, High-salt (6 g kg^−1^).

## Discussion

### Effects of salt stress on the carbon metabolism of Tartary buckwheat

The activity of carbon metabolism-related enzymes in plants is an important index for measuring the intensity of carbon metabolism. SS and SPS are soluble enzymes in the cytoplasm. SS mainly plays a role in cell wall and starch synthesis process and a key role in sucrose degradation^33^. SPS plays an active role in inducing sucrose synthesis in plants, and its enzymatic activity can directly determine the content of soluble sugars and reflect the “source” supply capacity to some extent. In plants, sugars serve as metabolic resources and structural components of cells, and sugars undergo osmotic adjustment under various stress conditions^34–36^. Sucrose is the main source of carbon and energy for plant metabolism.

Plants initiate regulatory mechanisms under stress conditions, adjust sucrose content by changing SS and SPS activities, and promote carbohydrate redistribution to adapt to stress^37^. Low salt stress induces SS and SPS activities in plants, thereby enhancing the ability of crops to synthesize sucrose and soluble sugars, and the accumulation of carbohydrates maintains cell osmotic pressure and exerts a positive effect, thereby enhancing resistance and ensures normal growth. By contrast, high salt stress decreases SS and SPS activities and inhibits the synthesis and transport of sucrose in the leaves and plant growth and development^38,39^. Wang et al.^40^ found that exogenous glucose and sucrose contribute to the growth of triticale seedlings under salt stress and low salt stress can induce increases in SPS and SS activities. In the present study, low salt stress promoted increases in SPS and SS activities in Tartary buckwheat leaves (Table 3), consistent with the above results.

Liu et al.^3^ pointed out that salt stress causes an increase in soluble sugars content. Gao^41^ found that soluble sugars content in rice leaves gradually increases with the level of saline-alkali stress. The results of this experiment showed that salt stress promoted the increase of soluble sugars and sucrose content in Tartary buckwheat leaves, consistent with the above research results. The reason may be related to self-regulation under stress. Salt stress changes ion concentration in leaves, thereby increasing SS and SPS activities and directly promoting the accumulation of sucrose and soluble sugars. The accumulation of large amounts of sucrose inhibits SS activity by negative feedback, weakening the inhibitory ability of SS reverse regulation on soluble sugars synthesis pathway. SS activity directly promotes the accumulation of total soluble sugars, and the accumulation of soluble sugars content have a significant effect on improving plant resistance. In the results of the present experiment, the activity of SS and SPS decreased significantly with salt stress treatment period and salt concentration. Therefore, sucrose in the leaves tended to degrade sugar and glucose. However, it only accounted for a small part of soluble sugars^14^. Therefore, the hydrolysis of sucrose into small molecular carbohydrates may not be the main mechanism by which Tartary buckwheat resists salt stress.

### Effects of salt stress on nitrogen metabolism in Tartary buckwheat

Nitrogen is a macro-essential element in plants and participates to structure of chlorophyll, proteins, nucleic acids, and rubisco involved in CO_2_ assimilation and quaternary ammonium compounds related to stress tolerance^42^. Stress restricts assimilation and translocation by limiting uptake and transport of NO_3_^−^ to plants and impairs the activities and synthesis of enzymes involved in assimilation^43^. Depending on these changes, stress limits the development, growth, and fertility of plants^44^. Katiyar and Dubey^45^ showed that the endogenous NR activity of salt-tolerant rice varieties increases significantly in response to salt stress, whereas the endogenous NR activity of salt-sensitive varieties decreases significantly in response to salt stress. GOGAT and GS constitute to the GS/GOGAT pathway, which catalyzes NH_4_^+^ assimilation reaction. This pathway is the center of the whole nitrogen metabolism, and most of the nitrogen content in plants is assimilated by this pathway^46^. Liu et al.^47^ found that the activities of NR, GS, and GOGAT increases under mild saline-alkali stress and short stress time, which stimulates the absorption of nitrogen by sugar beet, thereby reducing the harm of stress. However, it decreases significantly with further increase in saline-alkali stress level or stress time. The results of the present study showed that the activities of NR and GOGAT in Tartary buckwheat leaves first increased and then decreased with increasing salt concentration, reaching the maximum in the S2 treatment and the minimum in the S6 treatment. These results are basically consistent with the results of Liu et al.^47^ and indicate that moderate salt stress can increase the activities of enzymes related to nitrogen metabolism in Tartary buckwheat leaves which through nitrogen metabolism enzymes that can reduce the oxidative stress response of Tartary buckwheat under salt stress and can thus improve the stress resistance of plants^2^.

Soluble protein is an important organic osmotic adjustment substance in plants and plays an important role in alleviating damage. Its content is closely related to plant nitrogen metabolism and senescence. Cai et al.^48^ found that the soluble protein content of sweet potato seedlings under salt stress increases first and then decreases with increasing salt concentration. Similar results were obtained in the current experiment. When S2 was treated, the soluble protein content in the leaves of Tartary buckwheat reached the maximum and then decreased with further increase in salt concentration. Decrease in soluble protein content in leaves under high salt stress may be related to the fact that the Tartary buckwheat samples were in a state of nitrogen deficiency for a long time under salt stress and the activity of proteolytic enzymes in plants increased and promoted proteolysis.

Nitrate-nitrogen and ammonium nitrogen are the two most important inorganic nitrogen compounds absorbed and utilized by plants. The results of this experiment showed that the content of nitrate-nitrogen and ammonium nitrogen in the leaves increased first and then decreased with increasing salt concentration, reached the maximum in the S2 treatment, decreased with further increase in salt concentration, and was lower than that in the control treatment. The results indicated that salt stress had a “low promotion and high inhibition” effect on the content of nitrate-nitrogen and ammonium nitrogen possibly due to the competitive absorption of Cl^−^ and NO_3_^−^ under salt stress or the inhibition of nitrate on transport.

### Effects of salt stress on yield of Tartary buckwheat

Meng et al.^49^ showed that the spike number, grain number per spike, and 1000-grain weight of wheat decreased under salt stress, and the decrease rate increased with salt concentration. Chen et al.^50^ showed that the spike number and 1000-grain weight of oats were greatly affected by salt stress, and the yield decreased. Zhang et al.^51^ found that salt stress significantly decreased 1000-grain weight of rice. Consistent with the results mentioned above, the present study showed that the grain number per plant, grain weight per plant, and 100-grain weight of Tartary buckwheat first increased and then decreased with the increasing salt concentration, indicating that low concentration of salt stress can promote the growth and yield of Tartary buckwheat. This may be due to promoted root growth and root activity of Tartary buckwheat under low salt stress (Table 1). These effects facilitate the absorption of water and nutrients in the soil tillage layer by Tartary buckwheat roots, improve the net photosynthetic rates of the above-ground leaves, prolong the photosynthesis time of leaves, and improve dry matter production^52,53^, thereby increasing final yield per plant (Table 6). The possible reason also is that because low salt stress can increase the activities of key enzymes in carbon and nitrogen metabolism in Tartary buckwheat and increase the synthesis, transport, and accumulation of organic compounds in plants, resulting in increased yield per plant. High salt stress inhibits photosynthesis and Tartary buckwheat growth and thus affects dry matter production, accumulation, transfer, and distribution and leads to a decrease in yield per plant.

## Conclusions

Low salt stress treatment (2 g kg^−1^) promoted root growth, improved root activity, significantly increased the content of carbon and nitrogen metabolites and related enzyme activities in the leaves of Tartary buckwheat, ensured the metabolic balance of cells, and improved the osmotic adjustment ability by accumulating osmotic adjustment substances, such as soluble sugars. As result, the tolerance of Tartary buckwheat and the yield per plant under salt stress increased. High concentration of salt treatment reduced the osmotic adjustment substances such as soluble sugars, destroyed the structure and function of cell membrane, accelerated senescence, affected the physiological metabolism and photosynthesis, and thus affects dry matter production and accumulation and leads to inhibited the growth of Tartary buckwheat. The effects of salt stress on the growth of Tartary buckwheat were low promotion and high inhibition. The tolerance of Tartary buckwheat to salt stress has a threshold, that is, 2 g kg^−1^.

## Supplementary Information

Supplementary Tables.

## Data Availability

The data that support this study are available in the article and accompanying online Supplementary material.
