# Supplementary material for: Effects of salt stress on root morphology, carbon and nitrogen metabolism, and yield of Tartary buckwheat
Source: Sci Rep. 2023 Aug 1;13:12483. doi: 10.1038/s41598-023-39634-0 (PMC10393950; doi:10.1038/s41598-023-39634-0)
Supplement: Supplementary file 1 — Supplementary Tables. [file 41598_2023_39634_MOESM1_ESM.docx]

**Appendix: the data from the growth chamber**

Table S1 Effects of salt stress on root morphology and root activity of Tartary buckwheat

| Item | Treatment | Seedling stage | Flowering stage | Grain filing stage | Maturate  stage |
| --- | --- | --- | --- | --- | --- |
| Root length  (cm) | CK | 21.79±2.23b | 45.30±1.18a | 81.31±2.69a | 93.45±2.20a |
|  | S2 | 29.82±2.26a | 46.72±1.71a | 84.97±3.71a | 95.78±2.77a |
|  | S4 | 15.63±1.42c | 43.52±2.26a | 35.63±2.42b | 27.46±1.78b |
|  | S6 | 14.03±1.01c | 40.57±1.47b | 31.17±1.63b | 24.37±1.76b |
| Root surface area  (cm^2^) | CK | 4.38±0.17a | 6.56±0.15b | 14.71±2.46a | 19.59±0.77a |
|  | S2 | 5.27±0.10a | 9.70±0.19a | 16.16±1.85a | 20.24±1.33a |
|  | S4 | 3.22±0.12b | 5.81±0.17b | 13.84±1.58b | 18.43±0.86b |
|  | S6 | 2.78±0.13b | 5.15±0.23b | 13.26±1.05b | 17.47±1.05b |
| Root volume (cm^3^) | CK | 0.28±0.03a | 0.85±0.02a | 1.64±0.21a | 3.42±0.16a |
|  | S2 | 0.31±0.02a | 0.99±0.04a | 1.76±0.15a | 3.72±0.15a |
|  | S4 | 0.26±0.01a | 0.56±0.03b | 1.45±0.20b | 2.86±0.19b |
|  | S6 | 0.25±0.02a | 0.48±0.05b | 1.44±0.18b | 2.64±0.13b |
| Average diameter  (mm) | CK | 0.60±0.08a | 0.69±0.04b | 0.90±0.04b | 0.86±0.07a |
|  | S2 | 0.74±0.03a | 0.92±0.01a | 1.00±0.05a | 0.93±0.02a |
|  | S4 | 0.54±0.03a | 0.61±0.03b | 0.69±0.07b | 0.62±0.04b |
|  | S6 | 0.47±0.05b | 0.54±0.02b | 0.65±0.04b | 0.55±0.03b |
| Root activity (μg (gh) ^-1^) | CK | 6.64±0.17b | 6.21±0.16a | 2.12±0.11a | 1.99±0.16a |
|  | S2 | 8.52±0.18a | 6.80±0.17a | 2.53±0.12a | 2.10±0.10a |
|  | S4 | 6.37±0.25b | 6.05±0.23a | 1.74±0.09b | 0.86±0.07b |
|  | S6 | 5.89±0.24b | 5.63±0.29a | 1.65±0.07b | 0.76±0.04b |

Note: Different letters indicate statistical significance at the *P＜*0.05 level within the same column.

CK: control (0g kg^-1^), S2: low-salt (2g kg^-1^), S4: medium-salt (4g kg^-1^), S6: high-salt (6g kg^-1^).

Table S2 Effects of salt stress on carbon metabolism related substances in Tartary buckwheat

| Item | Treatment | Seedling stage | Flowering stage | Grain filing stage | Maturate stage |
| --- | --- | --- | --- | --- | --- |
| Soluble sugars  (mg g^-1^) | CK | 62.10±1.49d | 97.31±1.21d | 226.81±16.18c | 113.34±8.69d |
|  | S2 | 70.11±1.58c | 120.34±9.81c | 242.83±16.32b | 130.57±8.26c |
|  | S4 | 81.05±2.54b | 136.35±6.10b | 255.85±13.83b | 150.47±6.85b |
|  | S6 | 90.18±2.25a | 158.39±8.15a | 270.86±10.52a | 170.75±8.41a |
| Sucrose (mg g^-1^) | CK | 27.14±1.52d | 45.30±2.03d | 152.34±11.02d | 38.35±1.76d |
|  | S2 | 35.14±1.86c | 60.76±2.35c | 163.93±13.46c | 44.93±2.62c |
|  | S4 | 42.39±2.24b | 72.60±2.58b | 171.79±10.41b | 51.39±2.75b |
|  | S6 | 48.15±1.57a | 86.37±1.06a | 178.81±10.44a | 62.41±2.39a |

Note: Different letters indicate statistical significance at the *P＜*0.05 level within the same column.

CK: control (0g kg^-1^), S2: low-salt (2g kg^-1^), S4: medium-salt (4g kg^-1^), S6: high-salt (6g kg^-1^).

Table S3 Effects of salt stress on enzyme activities related to carbon metabolism in Tartary buckwheat

| Item | Treatment | Seedling stage | Flowering stage | Grain filing stage | Maturate stage |
| --- | --- | --- | --- | --- | --- |
| Invertase  (mg g^-1^ h^-1^) | CK | 4.51±0.13b | 8.78±0.10ab | 17.45±0.93ab | 9.33±0.15b |
|  | S2 | 4.88±0.16a | 8.93±0.17a | 17.69±0.67a | 9.67±0.19a |
|  | S4 | 4.21±0.12c | 8.58±0.15b | 17.17±0.60b | 9.07±0.14c |
|  | S6 | 4.01±0.16c | 8.27±0.25c | 16.93±0.92c | 8.83±0.16d |
| Amylase  (mg g^-1^ h^-1^) | CK | 1.32±0.11b | 2.47±0.16b | 6.59±0.21b | 2.67±0.15b |
|  | S2 | 1.46±0.10a | 2.69±0.18a | 6.90±0.18a | 2.83±0.13a |
|  | S4 | 1.29±0.07bc | 2.33±0.12bc | 6.45±0.31b | 2.61±0.09b |
|  | S6 | 1.25±0.04c | 2.17±0.13c | 6.18±0.16c | 2.26±0.14c |
| Sucrose synthase  (SS, mg g^-1^ h^-1^) | CK | 16.71±1.03b | 25.59±1.02b | 39.42±1.44b | 32.50±1.86ab |
|  | S2 | 18.50±1.01a | 27.28±1.39a | 41.71±2.29a | 34.38±1.80a |
|  | S4 | 14.32±1.04c | 22.75±1.27c | 36.54±1.52c | 30.69±1.18b |
|  | S6 | 13.54±1.15d | 19.63±0.96d | 34.46±1.33d | 29.48±1.47c |
| Sucrose phosphate synthase  (SPS, mg g^-1^ h^-1^) | CK | 12.66±0.73b | 20.78±1.58b | 33.46±2.19b | 27.42±1.48b |
|  | S2 | 14.75±0.88a | 22.38±1.32a | 36.75±2.52a | 29.48±1.67a |
|  | S4 | 9.28±0.13c | 18.79±1.81c | 31.97±2.10c | 25.54±2.02c |
|  | S6 | 7.86±0.15d | 14.65±1.13d | 29.89±1.88d | 23.69±1.68d |

Note: Different letters indicate statistical significance at the *P＜*0.05 level within the same column.

CK: control (0g kg^-1^), S2: low-salt (2g kg^-1^), S4: medium-salt (4g kg^-1^), S6: high-salt (6g kg^-1^).

Table S4 Effects of salt stress on nitrogen metabolism related substances in Tartary buckwheat

| Item | Treatment | Seedling stage | Flowering stage | Grain filing stage | Maturate stage |
| --- | --- | --- | --- | --- | --- |
| Nitrate nitrogen  (mg g^-1^) | CK | 81.56±2.27b | 113.47±8.93a | 144.74±10.22b | 97.77±5.16b |
|  | S2 | 88.60±1.86a | 119.85±9.43a | 151.38±9.67a | 102.85±5.39a |
|  | S4 | 79.53±1.41b | 108.79±8.22b | 138.29±9.84c | 90.27±3.76c |
|  | S6 | 72.62±2.10c | 102.20±7.18c | 131.46±10.59d | 82.67±4.47d |
| Ammonium nitrogen  (mg g^-1^) | CK | 97.72±2.09b | 109.93±9.29b | 128.93±8.62b | 91.36±6.85ab |
|  | S2 | 105.45±4.21a | 118.78±9.64a | 136.11±10.86a | 94.58±5.26a |
|  | S4 | 82.40±2.38c | 101.02±9.50c | 111.11±9.24c | 86.75±6.63b |
|  | S6 | 68.38±3.06d | 82.68±6.14d | 97.01±5.37d | 81.50±7.47c |
| Soluble protein  (mg g^-1^) | CK | 18.36±0.71b | 72.61±6.74b | 58.39±2.11b | 46.68±2.15b |
|  | S2 | 18.98±0.76a | 73.52±5.27a | 61.02±2.61a | 47.76±2.08a |
|  | S4 | 17.78±0.72c | 71.18±6.67c | 58.08±2.06bc | 45.16±1.14c |
|  | S6 | 17.35±0.64c | 70.01±6.59d | 57.60±2.13c | 44.91±1.63d |

Note: Different letters indicate statistical significance at the *P＜*0.05 level within the same column.

CK: control (0g kg^-1^), S2: low-salt (2g kg^-1^), S4: medium-salt (4g kg^-1^), S6: high-salt (6g kg^-1^).

Table S5 Effects of salt stress on enzyme activities related to nitrogen metabolism in Tartary buckwheat

| Item | Treatment | Seedling stage | Flowering  stage | Grain filing stage | Maturate stage |
| --- | --- | --- | --- | --- | --- |
| Nitrate Reductase (NR, mg g^-1^ h^-1^ ) | CK | 13.56±0.91ab | 45.13±1.12ab | 70.72±1.98b | 22.80±0.84b |
|  | S2 | 13.90±0.87a | 45.96±1.39a | 71.14±1.09a | 23.55±0.73a |
|  | S4 | 13.42±0.41b | 44.85±1.17b | 70.05±1.13c | 22.16±0.57c |
|  | S6 | 12.99±0.92c | 44.02±1.01c | 69.58±2.04d | 21.86±0.99d |
| Glutamate synthase  (GOGAT, mg g^-1^ h^-1^) | CK | 13.32±0.64b | 45.69±1.41b | 68.37±1.16b | 23.85±1.08b |
|  | S2 | 13.95±0.93a | 46.27±1.26a | 69.31±1.11a | 24.34±1.20a |
|  | S4 | 12.81±0.69c | 45.33±1.02c | 67.76±1.48bc | 23.32±1.74c |
|  | S6 | 12.30±0.73d | 45.04±0.72c | 67.10±1.27c | 22.97±1.29c |
| Glutamate dehydrogenase  (GDH, mg g^-1^ h^-1^) | CK | 13.34±0.66c | 23.79±1.78d | 26.07±1.03d | 14.10±0.62d |
|  | S2 | 13.38±0.77c | 24.96±1.39c | 27.66±0.79c | 14.51±0.74c |
|  | S4 | 14.36±0.74b | 25.48±1.53b | 28.01±0.86b | 15.34±0.50b |
|  | S6 | 14.97±0.79a | 26.26±1.05a | 28.94±1.08a | 15.98±0.81a |

Note: Different letters indicate statistical significance at the *P＜*0.05 level within the same column.

CK: control (0g kg^-1^), S2: low-salt (2g kg^-1^), S4: medium-salt (4g kg^-1^), S6: high-salt (6g kg^-1^).

Table S6 Effects of salt stress on yield of Tartary buckwheat

| Treatment | Grain number per plant | Hundred grain weight(g) | Grain weight per plant(g) |
| --- | --- | --- | --- |
| CK | 25.75±0.73a | 0.41±0.02a | 2.48±0.07a |
| S2 | 25.67±0.69a | 0.41±0.03a | 2.38±0.10b |
| S4 | 24.50±0.81a | 0.34±0.02ab | 2.24±0.08c |
| S6 | 21.67±0.75b | 0.32±0.02b | 2.23±0.05c |

Note: Different letters indicate statistical significance at the *P＜*0.05 level within the same column.

CK: control (0g kg^-1^), S2: low-salt (2g kg^-1^), S4: medium-salt (4g kg^-1^), S6: high-salt (6g kg^-1^).
